# Correction: Computed tomography-derived radiomic signature of head and neck squamous cell carcinoma (peri)tumoral tissue for the prediction of locoregional recurrence and distant metastasis after concurrent chemo-radiotherapy

**DOI:** 10.1371/journal.pone.0237048

**Published:** 2020-07-28

**Authors:** Simon Keek, Sebastian Sanduleanu, Frederik Wesseling, Reinout de Roest, Michiel van den Brekel, Martijn van der Heijden, Conchita Vens, Calareso Giuseppina, Lisa Licitra, Kathrin Scheckenbach, Marije Vergeer, C. René Leemans, Ruud H. Brakenhoff, Irene Nauta, Stefano Cavalieri, Henry C. Woodruff, Tito Poli, Ralph Leijenaar, Frank Hoebers, Philippe Lambin

The affiliation for the fifteenth author is incorrect. Stefano Cavalieri is not affiliated with #8 but with #9: Fondazione IRCCS Istituto nazionale dei tumori, Head and Neck Medical Oncology Unit, Milan, Italy.
