# Supplementary material for: Computed tomography-derived radiomic signature of head and neck squamous cell carcinoma (peri)tumoral tissue for the prediction of locoregional recurrence and distant metastasis after concurrent chemo-radiotherapy
Source: PLoS One. 2020 May 22;15(5):e0232639. doi: 10.1371/journal.pone.0232639 (PMC7244120; doi:10.1371/journal.pone.0232639)
Supplement: S1 Appendix — (DOCX) [file pone.0232639.s001.docx]

**Appendix A: Datasets, imaging parameters and missing data**

| **Dataset**  **(center)** | **Nr. patients** | **Nr. With contrast enhanced CT (CECT)** | **Original slice thickness (mm, median)** | **Original pixel spacing (mm, median)** |
| --- | --- | --- | --- | --- |
| **DESIGN (training)** |  |  |  |  |
| VUMC | 88 | 88 | 2.5 mm | 0.96x0.96 |
| UMCU | 81 | 81 | 2.0 mm | 0.98x0.98 |
| NKI | 102 | 102 | 3.0 mm | 0.98x0.98 |
| MAASTRO | 30 | 30 | 3.0 mm | 0.98x0.98 |
| **BD2DECIDE**  **(validation)** |  |  |  |  |
| VUMC | 55 | 55 | 2.5 mm | 0.96x0.96 |
| UDUS | 10 | 10 | 3.0 mm | 4.60x4.60 |
| INT | 11 | 11 | 2.5 mm | 4.60x4.60 |
| MAASTRO | 40 | 40 | 3.0 mm | 0.98x0.98 |

Table 1. Datasets and imaging parameters in survival analysis


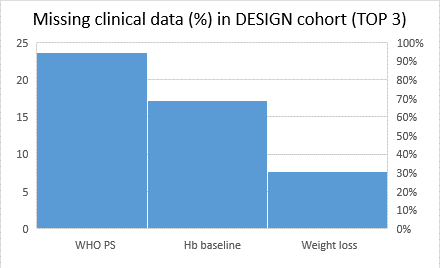


Table 2. Top 3 missing variables in training (DESIGN) cohort.
