# Supplementary material for: Computed tomography-derived radiomic signature of head and neck squamous cell carcinoma (peri)tumoral tissue for the prediction of locoregional recurrence and distant metastasis after concurrent chemo-radiotherapy
Source: PLoS One. 2020 May 22;15(5):e0232639. doi: 10.1371/journal.pone.0232639 (PMC7244120; doi:10.1371/journal.pone.0232639)
Supplement: S2 Appendix — (DOCX) [file pone.0232639.s002.docx]

**Appendix B: full names of ALL the ethics committees/institutional review boards that approved study**

-Stichting VU-VUmc (VU/VUmc), NL6, 53815211 established in De Boelelaan 1105, 1081 HV, Amsterdam (The Netherlands), represented by Prof. Johannes Brug, Director and Dean

-Heinrich-Heine Universitaetet Duesseldorf (UDUS), CF10657730442, established in Universitaetsstrasse 1, 40225 Duesseldorf (Germany), DE811222416), represented by Dr. Martin Goch, Chancellor

-Fondazione IRCCS Instituto Nazionale dei tumori (INT), VIII/002398, established in Via Venezian 1, Milan 20133, Italy, IT0437635055, represented by mr. Enzo Lucchini, President

-Stichting Maastro Radiation Oncology MAASTRO Clinic (MAASTRO) NL6, 41070330, established in Dr. Tanslaan 12, Maastricht 6229 ET, The Netherlands, represented by Mrs. Maria Jacobs, Administration Chief

-Stichting het Nederlands Kanker Instituut-Antoni van Leeuwenhoek ziekenhuis (Netherlands Cancer Institute/ Antoni van Leeuwenhoek Hospital), established at Plesmanlaan 121 1066 CX Amsterdam, in this matter duly represented by Prof. R Medema, P.h.d, in his capacity of Scientific Director and Chairman of the Board

-Universitair Medisch Centrum Utrect, established at Heidelberglaan 100, 3584 CX Utrecht, in this matter duly represented by Prof. dr. ir. M.A. Viergever, Manager Research, and Mr. drs. H.K. Bouwer, Financila Manager

-Maastricht University, more specific its Faculty of Health, Medicine and Life Sciences, School of Oncology and Developmental Biology (GROW), having its principle office at Minderbroedersberg 4-6, 6211 LK Maastricht, The Netherlands, on behalf of the Executive Board represented by Prof Dr. Frans Ramaekers, Scientific Director GROW (third party, analysis of data).
