# Supplementary material for: Computed tomography-derived radiomic signature of head and neck squamous cell carcinoma (peri)tumoral tissue for the prediction of locoregional recurrence and distant metastasis after concurrent chemo-radiotherapy
Source: PLoS One. 2020 May 22;15(5):e0232639. doi: 10.1371/journal.pone.0232639 (PMC7244120; doi:10.1371/journal.pone.0232639)
Supplement: S3 Appendix — (DOCX) [file pone.0232639.s003.docx]

**Appendix C: TRIPOD adherence data extraction checklist**
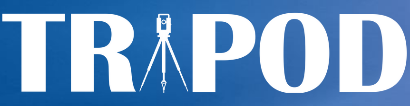


Prediction Model Development and Validation

| **B. TRIPOD ITEMS** | | | | |  | |  | |  | |  | |
| --- | --- | --- | --- | --- | --- | --- | --- | --- | --- | --- | --- | --- |
|  | |  |  | | **[D]**  **Development** | | **[V]**  **External validation** | | **[IV]**  **Incremental value** | | **[D+V]**  **Development and external validation (of same model)** | |
| **Title and abstract**  ***It is suggested to score items 1 and 2 (Title and Abstract) after scoring items 3 to 22, as only after reading the whole publication it can be judged whether the reporting in the title and abstract is complete.*** | | | | | | | | | | | | |
| **Title** | **1** | | **Identify the study as developing and/or validating a multivariable prediction model, the target population, and the outcome to be predicted.** | | **Score 1 if all extraction items are scored as “Y”** | | **Score 1 if all extraction items are scored as “Y”** | | **Score 1 if all extraction items are scored as “Y”** | | **Score 1 if all extraction items are scored as “Y”** | |
|  | i | | The words developing/development, validation/validating, incremental/added value (or synonyms) are reported in the title | | 0 | | 0 | | 0 | | 0 | |
|  | ii | | The words prediction, risk prediction, prediction model, risk models, prognostic models, prognostic indices, risk scores (or synonyms) are reported in the title | | 1 | | 1 | | 0 | | 1 | |
|  | iii | | The target population is reported in the title | | 1 | | 1 | | 0 | | 0 | |
|  | iv | | The outcome to be predicted is reported in the title | | 1 | | 1 | | 0 | | 0 | |
| **Abstract** | **2** | | **Provide a summary of objectives, study design, setting, participants, sample size, predictors, outcome, statistical analysis, results, and conclusions.** | | **Score 1 if all extraction items are scored as “Y” or “NA”** | | **Score 1 if all extraction items are scored as “Y” or “NA”** | | **Score 1 if all extraction items are scored as “Y” or “NA”** | | **Score 1 if all extraction items are scored as “Y” or “NA”** | |
|  | i | | The objectives are reported in the abstract | | 1 | | 1 | | 0 | | 0 | |
|  | ii | | Sources of data are reported in the abstract *E.g. Prospective cohort, registry data, RCT data.* | | 1 | | 1 | | 0 | | 0 | |
|  | iii | | The setting is reported in the abstract *E.g. Primary care, secondary care, general population, adult care, or paediatric care. The setting should be reported for both the development and validation datasets, if applicable.* | | 0 | | 0 | | 0 | | 0 | |
|  | iv | | A general definition of the study participants is reported in the abstract *E.g. patients with suspicion of certain disease, patients with a specific disease, or general eligibility criteria.* | | 1 | | 1 | | 0 | | 1 | |
|  | v | | The overall sample size is reported in the abstract | | 1 | | 1 | | 0 | | 1 | |
|  | vi | | The number of events (or % outcome together with overall sample size) is reported in the abstract *If a continuous outcome was studied, score Not applicable.* | | 0 | | 0 | | 0 | | 0 | |
|  | vii | | Predictors included in the final model are reported in the abstract. For validation studies of well-known models, at least the name/acronym of the validated model is reported *Broad descriptions are sufficient, e.g. ‘all information from patient history and physical examination’. Check in the main text whether all predictors of the final model are indeed reported in the abstract.* | | 1 | | 1 | | 1 | | 1 | |
|  | viii | | The outcome is reported in the abstract | | 1 | | 1 | | 1 | | 1 | |
|  | ix | | Statistical methods are described in the abstract *For model development, at least the type of statistical model should be reported. For validation studies a quote like “model’s discrimination and calibration was assessed” is considered adequate. If done, methods of updating should be reported.* | | 1 | | 1 | | 1 | | 1 | |
|  | x | | Results for model discrimination are reported in the abstract *This should be reported separately for development and validation if a study includes both development and validation.* | | 1 | | 1 | | 1 | | 1 | |
|  | xi | | Results for model calibration are reported in the abstract *This should be reported separately for development and validation if a study includes both development and validation.* | | 0 | | 0 | | 0 | | 0 | |
|  | xii | | Conclusions are reported in the abstract *In publications addressing both model development and validation, there is no need for separate conclusions for both; one conclusion is sufficient.* | | 1 | | 1 | | 1 | | 1 | |
| **Background and objectives** | **3a** | | **Explain the medical context (including whether diagnostic or prognostic) and rationale for developing or validating the multivariable prediction model, including references to existing models.** | | **Score 1 if both extraction items are scored as “Y”** | | **Score 1 if both extraction items are scored as “Y”** | | **Score 1 if both extraction items are scored as “Y”** | | **Score 1 if both extraction items are scored as “Y”** | |
|  | i | | The background and rationale are presented | | 1 | | 1 | | 1 | | 1 | |
|  | ii | | Reference to existing models is included (or stated that there are no existing models) | | 1 | | 1 | | 1 | | 1 | |
|  | **3b** | | **Specify the objectives, including whether the study describes the development or validation of the model or both.** | | **Score 1 if extraction item is scored as “Y”** | | **Score 1 if extraction item is scored as “Y”** | | **Score 1 if extraction item is scored as “Y”** | | **Score 1 if extraction item is scored as “Y”** | |
|  | i | | It is stated whether the study describes development and/or validation and/or incremental (added) value | | 1 | | 1 | | 1 | | 1 | |
| **Methods** | | | | |  | |  | |  | |  | |
| **Source of data** | **4a** | | **Describe the study design or source of data (e.g., randomized trial, cohort, or registry data), separately for the development and validation data sets, if applicable.** | | **Score 1 if extraction item is scored as “Y”** | | **Score 1 if extraction item is scored as “Y”** | | **Score 1 if extraction item is scored as “Y”** | | **Score 1 if extraction item is scored as “Y”** | |
|  | i | | The study design/source of data is described *E.g. Prospectively designed, existing cohort, existing RCT, registry/medical records, case control, case series. This needs to be explicitly reported; reference to this information in another article alone is insufficient.* | | 1 | | 1 | | 1 | | 1 | |
|  | **4b** | | **Specify the key study dates, including start of accrual; end of accrual; and, if applicable, end of follow-up.** | | **Score 1 if all extraction items are scored as ”Y”, “NA”, or “R”** | | **Score 1 if all extraction items are scored as ”Y”, “NA”, or “R”** | | **Score 1 if all extraction items are scored as ”Y”, “NA”, or “R”** | | **Score 1 if all extraction items are scored as ”Y”, “NA”, or “R”** | |
|  | i | | The starting date of accrual is reported | | 1 | | 1 | | NA | | 1 | |
|  | ii | | The end date of accrual is reported | | 1 | | 1 | | NA | | 1 | |
|  | iii | | The length of follow-up and prediction horizon/time frame are reported, if applicable *E.g. “Patients were followed from baseline for 10 years“ and “10-year prediction of…”; notably for prognostic studies with long term follow-up. If this is not applicable for an article (i.e. diagnostic study or no follow-up), then score Not applicable.* | | NA | | NA | | NA | | NA | |
| **Participants** | **5a** | | **Specify key elements of the study setting (e.g., primary care, secondary care, general population) including number and location of centres.** | | **Score 1 if all extraction items are scored as “Y” or “R”** | | **Score 1 if all extraction items are scored as “Y” or “R”** | | **Score 1 if all extraction items are scored as “Y” or “R”** | | **Score 1 if all extraction items are scored as “Y” or “R”** | |
|  | i | | The study setting is reported (e.g. primary care, secondary care, general population) *E.g.: ‘surgery for endometrial cancer patients’ is considered to be enough information about the study setting.* | | 1 | | 1 | | 1 | | 1 | |
|  | ii | | The number of centres involved is reported *If the number is not reported explicitly, but can be concluded from the name of the centre/centres, or if clearly a single centre study, score Yes.* | | 1 | | 1 | | 1 | | 1 | |
|  | iii | | The geographical location (at least country) of centres involved is reported *If no geographical location is specified, but the location can be concluded from the name of the centre(s), score Yes.* | | 1 | | 1 | | 1 | | 1 | |
|  | **5b** | | **Describe eligibility criteria for participants.** | | **Score 1 if extraction item is scored as “Y”** | | **Score 1 if extraction item is scored as “Y”** | | **Score 1 if extraction item is scored as “Y”** | | **Score 1 if extraction item is scored as “Y”** | |
|  | i | | In-/exclusion criteria are stated *These should explicitly be stated. Reasons for exclusion only described in a patient flow is not sufficient.* | | 1 | | 1 | | 0 | | 1 | |
|  | **5c** | | **Give details of treatments received, if relevant.** | | **Score 1 if extraction item is scored as “Y”; score *Not applicable* if extraction item is scored as “NA”** | | **Score 1 if extraction item is scored as “Y”; score *Not applicable* if extraction item is scored as “NA”** | | **Score 1 if extraction item is scored as “Y”; score *Not applicable* if extraction item is scored as “NA”** | | **Score 1 if extraction item is scored as “Y”; score *Not applicable* if extraction item is scored as “NA”** | |
|  | i | | Details of any treatments received are described  *This item is notably for prognostic modelling studies and is about treatment at baseline or during follow-up. The ‘if relevant’ judgment of treatment requires clinical knowledge and interpretation.*  *If you are certain that treatment was not relevant, e.g. in some diagnostic model studies, score Not applicable.* | | 1 | | 1 | | 1 | | 1 | |
| **Outcome** | **6a** | | **Clearly define the outcome that is predicted by the prediction model, including how and when assessed.** | | **Score 1 if all extraction items are scored as “Y” or “R”** | | **Score 1 if all extraction items are scored as “Y” or “R”** | | **Score 1 if all extraction items are scored as “Y” or “R”** | | **Score 1 if all extraction items are scored as “Y” or “R”** | |
|  | i | | The outcome definition is clearly presented *This should be reported separately for development and validation if a publication includes both.* | | 1 | | 1 | | 1 | | 1 | |
|  | ii | | It is described how outcome was assessed (including all elements of any composite, for example CVD [e.g. MI, HF, stroke])*.* | | 1 | | 1 | | 1 | | 1 | |
|  | iii | | It is described when the outcome was assessed (time point(s) since T0) | | 1 | | 1 | | 1 | | 1 | |
|  | **6b** | | **Report any actions to blind assessment of the outcome to be predicted.** | | **Score 1 if extraction item is scored as “Y”** | | **Score 1 if extraction item is scored as “Y”** | | **Score 1 if extraction item is scored as “Y”** | | **Score 1 if extraction item is scored as “Y”** | |
|  | i | | Actions to blind assessment of outcome to be predicted are reported *If it is clearly a non-issue (e.g. all-cause mortality or an outcome not requiring interpretation), score Yes. In all other instances, an explicit mention is expected.* | | 1 | | 1 | | 1 | | 1 | |
| **Predictors** | **7a** | | **Clearly define all predictors used in developing or validating the multivariable prediction model, including how and when they were measured.** | | **Score 1 if all extraction items are scored as “Y” or “R”** | | **Score 1 if all extraction items are scored as “Y” or “R”** | | **Score 1 if all extraction items are scored as “Y” or “R”** | | **Score 1 if all extraction items are scored as “Y” or “R”** | |
|  | i | | All predictors are reported *For development, “all predictors” refers to all predictors that potentially could have been included in the ‘final’ model (including those considered in any univariable analyses). For validation, “all predictors” means the predictors in the model being evaluated.* | | 1 | | 1 | | 1 | | 1 | |
|  | ii | | Predictor definitions are clearly presented | | 1 | | 1 | | 1 | | 1 | |
|  | iii | | It is clearly described how the predictors were measured | | 1 | | 1 | | 1 | | 1 | |
|  | iv | | It is clearly described when the predictors were measured | | 1 | | 1 | | 1 | | 1 | |
|  | **7b** | | **Report any actions to blind assessment of predictors for the outcome and other predictors.** | | **Score 1 if both extraction items are scored as “Y”** | | **Score 1 if both extraction items are scored as “Y”** | | **Score 1 if both extraction items are scored as “Y”** | | **Score 1 if both extraction items are scored as “Y”** | |
|  | i | | It is clearly described whether predictor assessments were blinded for outcome *For predictors for which it is clearly a non-issue (e.g. automatic blood pressure measurement, age, sex) and for instances where the predictors were clearly assessed before outcome assessment, score Yes. For all other predictors an explicit mention is expected.* | | 0 | | 0 | | 0 | | 0 | |
|  | ii | | It is clearly described whether predictor assessments were blinded for the other predictors | | 0 | | 0 | | 0 | | 0 | |
| **Sample size** | **8** | | **Explain how the study size was arrived at.** | | **Score 1 if extraction item is scored as “Y”** | | **Score 1 if extraction item is scored as “Y”** | | **Score 1 if extraction item is scored as “Y”** | | **Score 1 if extraction item is scored as “Y”** | |
|  | i | | It is explained how the study size was arrived at *Is there any mention of sample size, e.g. whether this was done on statistical grounds or practical/logistical grounds (e.g. an existing study cohort or data set of a RCT was used)?* | | 1 | | 1 | | 1 | | 1 | |
| **Missing data** | **9** | | **Describe how missing data were handled (e.g., complete-case analysis, single imputation, multiple imputation) with details of any imputation method.** | | **Score 1 if all extraction items are scored as “Y” or “NA”** | | **Score 1 if all extraction items are scored as “Y” or “NA”** | | **Score 1 if all extraction items are scored as “Y” or “NA”** | | **Score 1 if all extraction items are scored as “Y” or “NA”** | |
|  | i | | The method for handling missing data (predictors and outcome) is mentioned *E.g. Complete case (explicit mention that individuals with missing values have been excluded), single imputation, multiple imputation, mean/median imputation.*  *If there is no missing data, there should be an explicit mention that there is no missing data for all predictors and outcome. If so, score Yes. If it is unclear whether there is missing data (from e.g. the reported methods or results), score No. If it is clear there is missing data, but the method for handling missing data is unclear, score No.* | | 1 | | 1 | | 1 | | 1 | |
|  | ii | | If missing data were imputed, details of the software used are given  *When under 9i explicit mentioning of no missing data, complete case analysis or no imputation applied, score Not applicable.* | | 1 | | 1 | | 1 | | 1 | |
|  | iii | | If missing data were imputed, a description of which variables were included in the imputation procedure is given.  *When under 9i explicit mentioning of no missing data, complete case analysis or no imputation applied, score Not applicable.* | | 1 | | 1 | | 1 | | 1 | |
|  | iv | | If multiple imputation was used, the number of imputations is reported  *When under 9i explicit mentioning of no missing data, complete case analysis or no imputation applied, score Not applicable.* | | NA | | NA | | NA | | NA | |
| **Statistical analysis methods** | **10a** | | **Describe how predictors were handled in the analyses.** | | **Score 1 if all extraction items are scored as “Y” or “NA”** | | **Not applicable** | | **Score 1 if all extraction items are scored as “Y” or “NA”** | | **Score 1 if all extraction items are scored as “Y” or “NA”** | |
|  | i | | For continuous predictors it is described whether they were modelled as linear, nonlinear (type of transformation specified) or categorized *A general statement is sufficient, no need to describe this for each predictor separately.  If no continuous predictors were reported, score Not applicable.* | | 1 | | Not applicable | | 1 | | 1 | |
|  | ii | | For categorical or categorized predictors, the cut-points were reported *If no categorical or categorized predictors were reported, score Not applicable.* | | NA | | Not applicable | | NA | | NA | |
|  | iii | | For categorized predictors the method to choose the cut-points was clearly described *If no categorized predictors, score Not applicable.* | | NA | | Not applicable | | NA | | NA | |
|  | **10b** | | **Specify type of model, all model-building procedures (including any predictor selection), and method for internal validation.** | | **Score 1 if all extraction items are scored as “Y” or “NA”** | | **Not applicable** | | **Score 1 if all extraction items are scored as “Y” or “NA”** | | **Score 1 if all extraction items are scored as “Y” or “NA”** | |
|  | i | | The type of statistical model is reported *E.g. Logistic, Cox, other regression model (e.g. Weibull, ordinal), other statistical modelling (e.g. neural network)* | | 1 | | Not applicable | | 1 | | 1 | |
|  | ii | | The approach used for predictor selection before modelling is described *‘Before modelling’ means before any univariable or multivariable analysis of predictor-outcome associations. If no predictor selection before modelling is done, score Not applicable. If it is unclear whether predictor selection before modelling is done, score No. If it is clear there was predictor selection before modelling but the method was not described, score No.* | | 1 | | Not applicable | | 1 | | 1 | |
|  | iii | | The approach used for predictor selection during modelling is described  *E.g. Univariable analysis, stepwise selection, bootstrap, Lasso.* *‘During modelling’ includes both univariable or multivariable analysis of predictor-outcome associations.  If no predictor selection during modelling is done (so-called full model approach), score Not applicable. If it is unclear whether predictor selection during modelling is done, score No.  If it is clear there was predictor selection during modelling but the method was not described, score No.* | | 1 | | Not applicable | | 1 | | 1 | |
|  | iv | | Testing of interaction terms is described  *If it is explicitly mentioned that interaction terms were not addressed in the prediction model, score Yes.*  *If interaction terms were included in the prediction model, but the testing is not described, score No.* | | 0 | | Not applicable | | 0 | | 0 | |
|  | v | | Testing of the proportionality of hazards in survival models is described *If no proportional hazard model is used, score Not applicable.* | | 1 | | Not applicable | | 1 | | 1 | |
|  | vi | | Internal validation is reported  *E.g. Bootstrapping, cross validation, split sample.*  *If the use of internal validation is clearly a non-issue (e.g. in case of very large data sets), score Yes. For all other situations an explicit mention is expected.* | | 1 | | Not applicable | | 1 | | 1 | |
|  | **10c** | | **For validation, describe how the predictions were calculated.** | | **Not applicable** | | **Score 1 if extraction item is scored as “Y”** | | **Score 1 if extraction item is scored as “Y”** | | **Score 1 if extraction item is scored as “Y”** | |
|  | i. | | It is described how predictions for individuals (in the validation set) were obtained from the model being validated  *E.g. Using the original reported model coefficients with or without the intercept, and/or using updated or refitted model coefficients, or using a nomogram, spreadsheet or web calculator.* | | Not applicable | | 1 | | 1 | | 1 | |
|  | **10d** | | **Specify all measures used to assess model performance and, if relevant, to compare multiple models.^^[[1]](#footnote-1)^^** *These should be described in the methods section of the paper (item 16 addresses the reporting of the results for model performance).* | | **Score 1 if extraction items 10di and 10dii are scored as “Y”** | | **Score 1 if extraction items 10di and 10dii are scored as “Y”** | | **Score 1 if all extraction items are scored as “Y”** | | **Score 1 if extraction items 10di and 10dii are scored as “Y”** | |
|  | i | | Measures for model discrimination are described *E.g. C-index / area under the ROC curve.* | | 1 | | 1 | | 1 | | 1 | |
|  | ii | | Measures for model calibration are described *E.g. calibration plot, calibration slope or intercept, calibration table, Hosmer Lemeshow test, O/E ratio.* | | 0 | | 0 | | 0 | | 0 | |
|  | iii | | Other performance measures are described  *E.g. R^2^, Brier score, predictive values, sensitivity, specificity, AUC difference, decision curve analysis, net reclassification improvement, integrated discrimination improvement, AIC.* | | 1 | | 1 | | 1 | | 1 | |
|  | **10e** | | **Describe any model updating (e.g., recalibration) arising from the validation, if done.** | | **Not applicable** | | **Score 1 if extraction item is scored as “Y”; score *Not applicable* if extraction item is scored as “NA”** | | **Score 1 if extraction item is scored as “Y”; score *Not applicable* if extraction item is scored as “NA”** | | **Score 1 if extraction item is scored as “Y”; score *Not applicable* if extraction item is scored as “NA”** | |
|  | i | | A description of model-updating is given *E.g. Intercept recalibration, regression coefficient recalibration, refitting the whole model, adding a new predictor*  *If updating was done, it should be clear which updating method was applied to score Yes.*  *If it is not explicitly mentioned that updating was applied in the study, score this item as ‘Not applicable’.* | | Not applicable | | NA | | NA | | NA | |
| **Risk groups** | **11** | | **Provide details on how risk groups were created, if done.** | | **Score 1 if extraction item is scored as “Y”; score *Not applicable* if extraction item is scored as “NA”** | | **Score 1 if extraction item is scored as “Y”; score *Not applicable* if extraction item is scored as “NA”** | | **Score 1 if extraction item is scored as “Y”; score *Not applicable* if extraction item is scored as “NA”** | | **Score 1 if extraction item is scored as “Y”; score *Not applicable* if extraction item is scored as “NA”** | |
|  | i | | If risk groups were created, risk group boundaries (risk thresholds) are specified  *Score this item separately for development and validation if a study includes both development and validation.*  *If risk groups were not created, score this item as not applicable.* | | NA | | NA | | NA | | NA | |
| **Development vs. validation** | **12** | | **For validation, identify any differences from the development data in setting, eligibility criteria, outcome and predictors.** | | **Not applicable** | | **Score 1 if extraction item is scored as “Y”** | | **Score 1 if extraction item is scored as “Y” or “NA”** | | **Score 1 if extraction item is scored as “Y”** | |
|  | i | | Differences or similarities in definitions with the development study are described *Mentioning of any differences in all four (setting, eligibility criteria, predictors and outcome) is required to score Yes.  If it is explicitly mentioned that there were no differences in setting, eligibility criteria, predictors and outcomes, score Yes.*  *For incremental value reports, in case additional predictors are not added to a previously developed prediction model but rather added to conventional predictors in a newly fitted model, score Not applicable.* | | Not applicable | | 0 | | 0 | | 0 | |
| **Results** | | | | |  | |  | |  | |  | |
| **Participants** | **13a** | | **Describe the flow of participants through the study, including the number of participants with and without the outcome and, if applicable, a summary of the follow-up time. A diagram may be helpful.** | **Score 1 if all extraction items are scored as “Y” or “NA”** | | **Score 1 if the extraction items are scored as “Y” or “NA”** | | **Score 1 if all extraction items are scored as “Y” or “NA”** | | **Score 1 if all extraction items are scored as “Y” or “NA”** | |  |
|  | i | | The flow of participants is reported | 1 | | 1 | | 1 | | 1 | |  |
|  | ii | | The number of participants with and without the outcome are reported *If outcomes are continuous, score Not applicable.* | 1 | | 1 | | 1 | | 1 | |  |
|  | iii | | A summary of follow-up time is presented *This notably applies to prognosis studies and diagnostic studies with follow-up as diagnostic outcome. If this is not applicable for an article (i.e. diagnostic study or no follow-up), then score Not applicable.* | 1 | | 1 | | 1 | | 1 | |  |
|  | **13b** | | **Describe the characteristics of the participants (basic demographics, clinical features, available predictors), including the number of participants with missing data for predictors and outcome.** | **Score 1 if all extraction items are scored as “Y”** | | **Score 1 if all extraction items are scored as “Y”** | | **Score 1 if all extraction items are scored as “Y”** | | **Score 1 if all extraction items are scored as “Y”** | |  |
|  | i | | Basic demographics are reported | 0 | | 0 | | 0 | | 0 | |  |
|  | ii | | Summary information is provided for all predictors included in the final developed/validated model | 1 | | 1 | | 1 | | 1 | |  |
|  | iii | | The number of participants with missing data for predictors is reported | 1 | | 1 | | 1 | | 1 | |  |
|  | iv | | The number of participants with missing data for the outcome is reported | 1 | | 1 | | 1 | | 1 | |  |
|  | **13c** | | **For validation, show a comparison with the development data of the distribution of important variables (demographics, predictors and outcome).** | **Not applicable** | | **Score 1 if all extraction items are scored as “Y”** | | **Score 1 if all extraction items are scored as “Y” or “NA”** | | **Score 1 if all extraction items are scored as “Y”** | |  |
|  | i | | Demographic characteristics (at least age and gender) of the validation study participants are reported along with those of the original development study  *For incremental value reports, in case additional predictors are not added to a previously developed prediction model but rather added to conventional predictors in a newly fitted model, score Not applicable.* | Not applicable | | 1 | | 1 | | 1 | |  |
|  | ii | | Distributions of predictors in the model of the validation study participants are reported along with those of the original development study  *For incremental value reports, in case additional predictors are not added to a previously developed prediction model but rather added to conventional predictors in a newly fitted model, score Not applicable.* | Not applicable | | 0 | | 0 | | 0 | |  |
|  | iii | | Outcomes of the validation study participants are reported along with those of the original development study  *For incremental value reports, in case additional predictors are not added to a previously developed prediction model but rather added to conventional predictors in a newly fitted model, score Not applicable.* | Not applicable | | 1 | | 1 | | 1 | |  |
| **Model development** | **14a** | | **Specify the number of participants and outcome events in each analysis.** | **Score 1 if both extraction items are scored as “Y” or “NA”** | | **Not applicable** | | **Score 1 if both extraction items are scored as “Y” or “NA”** | | **Score 1 if both extraction items are scored as “Y” or “NA”** | |  |
|  | i | | The number of participants in each analysis (e.g. in the analysis of each model if more than one model is developed) is specified | 1 | | Not applicable | | 1 | | 1 | |  |
|  | ii | | The number of outcome events in each analysis is specified (e.g. in the analysis of each model if more than one model is developed) *If outcomes are continuous, score Not applicable.* | 1 | | Not applicable | | 1 | | 1 | |  |
|  | **14b** | | **If done, report the unadjusted association between each candidate predictor and outcome.** | **Score 1 if extraction item is scored as “Y”; score *Not applicable* if extraction item is scored as “NA”** | | **Not applicable** | | **Score 1 if extraction item is scored as “Y”; score *Not applicable* if extraction item is scored as “NA”** | | **Score 1 if extraction item is scored as “Y”; score *Not applicable* if extraction item is scored as “NA”** | |  |
|  | i | | The unadjusted associations between each predictor and outcome are reported *If any univariable analysis is mentioned in the methods but not in the results, score No.*  *If nothing on univariable analysis (in methods or results) is reported, score this item as Not applicable.* | NA | | Not applicable | | NA | | NA | |  |
| **Model specification** | **15a** | | **Present the full prediction model to allow predictions for individuals (i.e., all regression coefficients, and model intercept or baseline survival at a given time point).** | **Score 1 if both extraction items are scored as “Y”** | | **Not applicable** | | **Score 1 if both extraction items are scored as “Y”** | | **Score 1 if both extraction items are scored as “Y”** | |  |
|  | i | | The regression coefficient (or a derivative such as hazard ratio, odds ratio, risk ratio) for each predictor in the model is reported | 0 | | Not applicable | | 0 | | 0 | |  |
|  | ii | | The intercept or the cumulative baseline hazard (or baseline survival) for at least one time point is reported | 1 | | Not applicable | | 1 | | 1 | |  |
|  | **15b** | | **Explain how to use the prediction model.** | **Score 1 if extraction item is scored as “Y”** | | **Not applicable** | | **Score 1 if extraction item is scored as “Y”** | | **Score 1 if extraction item is scored as “Y”** | |  |
|  | i | | An explanation (e.g. a simplified scoring rule, chart, nomogram of the model, reference to online calculator, or worked example) is provided to explain how to use the model for individualised predictions. | 1 | | Not applicable | | 1 | | 1 | |  |
| **Model performance** | **16** | | **Report performance measures (with confidence intervals) for the prediction model.^^[[2]](#footnote-2)^^** *These should be described in results section of the paper (item 10 addresses the reporting of the methods for model performance).* | **Score 1 if extraction itemsare scored as “Y”** | | **Score 1 if extraction items are scored as “Y”** | | **Score 1 if all extraction items are scored as “Y”** | | **Score 1 if extraction items are scored as “Y”** | |  |
|  | i | | A discrimination measure is presented *E.g. C-index / area under the ROC curve.* | 1 | | 1 | | 1 | | 1 | |  |
|  | ii | | The confidence interval (or standard error) of the discrimination measure is presented | 1 | | 1 | | 1 | | 1 | |  |
|  | iii | | Measures for model calibration are described *E.g. calibration plot, calibration slope or intercept, calibration table, Hosmer Lemeshow test, O/E ratio.* | 1 | | 1 | | 1 | | 1 | |  |
|  | iv | | Other model performance measures are presented *E.g. R^2^, Brier score, predictive values, sensitivity, specificity, AUC difference, decision curve analysis, net reclassification improvement, integrated discrimination improvement, AIC.* | 1 | | 1 | | 1 | | 1 | |  |
| **Model updating** | **17** | | **If done, report the results from any model updating (i.e., model specification, model performance, recalibration).**  *If updating was not done, score this TRIPOD item as ‘Not applicable’.* | **Not applicable** | | **Score 1 if all extraction items are scored as “Y”** | | **Not applicable** | | **Score 1 if all extraction items are scored as “Y”** | |  |
|  | i | | The updated regression coefficients for each predictor in the model are reported  *If model updating was described as ‘not needed’, score Yes.* | Not applicable | | NA | | Not applicable | | NA | |  |
|  | ii | | The updated intercept or cumulative baseline hazard or baseline survival (for at least one time point) is reported  *If model updating was described as ‘not needed’, score Yes.* | Not applicable | | NA | | Not applicable | | NA | |  |
|  | iii | | The discrimination of the updated model is reported | Not applicable | | NA | | Not applicable | | NA | |  |
|  | iv | | The confidence interval (or standard error) of the discrimination measure of the updated model is reported | Not applicable | | NA | | Not applicable | | NA | |  |
|  | v | | The calibration of the updated model is reported | Not applicable | | NA | | Not applicable | | NA | |  |
| **Discussion** | | | | |  | |  | |  | |  | |
| **Limitations** | **18** | | **Discuss any limitations of the study (such as nonrepresentative sample, few events per predictor, missing data).** | **Score 1 if extraction item is scored as “Y”** | | **Score 1 if extraction item is scored as “Y”** | | **Score 1 if extraction item is scored as “Y”** | | **Score 1 if extraction item is scored as “Y”** | |  |
|  | i | | Limitations of the study are discussed *Stating any limitation is sufficient.* | 1 | | 1 | | 1 | | 1 | |  |
| **Interpretation** | **19a** | | **For validation, discuss the results with reference to performance in the development data, and any other validation data.** | **Not applicable** | | **Score 1 if extraction item is scored as “Y”** | | **Score 1 if extraction item is scored as “Y”** | | **Score 1 if extraction item is scored as “Y”** | |  |
|  | i | | Comparison of results to reported performance in development studies and/or other validation studies is given | Not applicable | | 1 | | 1 | | 1 | |  |
|  | **19b** | | **Give an overall interpretation of the results considering objectives, limitations, results from similar studies and other relevant evidence.** | **Score 1 if extraction item is scored as “Y”** | | **Score 1 if extraction item is scored as “Y”** | | **Score 1 if extraction item is scored as “Y”** | | **Score 1 if extraction item is scored as “Y”** | |  |
|  | i | | An overall interpretation of the results is given | 1 | | 1 | | 1 | | 1 | |  |
| **Implications** | **20** | | **Discuss the potential clinical use of the model and implications for future research.** | **Score 1 if both extraction items are scored as “Y”** | | **Score 1 if both extraction items are scored as “Y”** | | **Score 1 if both extraction items are scored as “Y”** | | **Score 1 if both extraction items are scored as “Y”** | |  |
|  | i | | The potential clinical use is discussed  *E.g. an explicit description of the context in which the prediction model is to be used (e.g. to identify high risk groups to help direct treatment, or to triage patients for referral to subsequent care).* | 1 | | 1 | | 1 | | 1 | |  |
|  | ii | | Implications for future research are discussed *E.g. a description of what the next stage of investigation of the prediction model should be, such as ”We suggest further external validation”.* | 1 | | 1 | | 1 | | 1 | |  |
| **Other information** | | | | |  | |  | |  | |  | |
| **Supplementary information** | **21** | | **Provide information about the availability of supplementary resources, such as study protocol, web calculator, and data sets.** | **Not included in overall scoring** | | **Not included in overall scoring** | | **Not included in overall scoring** | | **Not included in overall scoring** | |  |
|  | i | | Information about supplementary resources is provided | 1 | | 1 | | 1 | | 1 | |  |
| **Funding** | **22** | | **Give the source of funding and the role of the funders for the present study.** | **Score 1 if both extraction items are scored as “Y”** | | **Score 1 if both extraction items are scored as “Y”** | | **Score 1 if both extraction items are scored as “Y”** | | **Score 1 if both extraction items are scored as “Y”** | |  |
|  | i | | The source of funding is reported or there is explicit mention that there was no external funding involved | 1 | | 1 | | 1 | | 1 | |  |
|  | ii | | The role of funders is reported or there is explicit mention that there was no external funding | 1 | | 1 | | 1 | | 1 | |  |
| Total Adherance | 23 | | Calculates the total Adherance ti the TRIPOD statement | 0.86 | | 0.86 | | 0.75 | | 0.80 | |  |

**Source:** <https://www.tripod-statement.org/>

1. [↑](#footnote-ref-1)
2. [↑](#footnote-ref-2)
